# Supplementary material for: Nanotoxicity of 2D Molybdenum Disulfide, MoS2, Nanosheets on Beneficial Soil Bacteria, Bacillus cereus and Pseudomonas aeruginosa
Source: Nanomaterials (Basel). 2021 May 31;11(6):1453. doi: 10.3390/nano11061453 (PMC8229097; doi:10.3390/nano11061453)
Supplement: Supplementary file 1 [file nanomaterials-11-01453-s001.zip › nanomaterials-1184889-supplementary.pdf]

Supplementary Materials

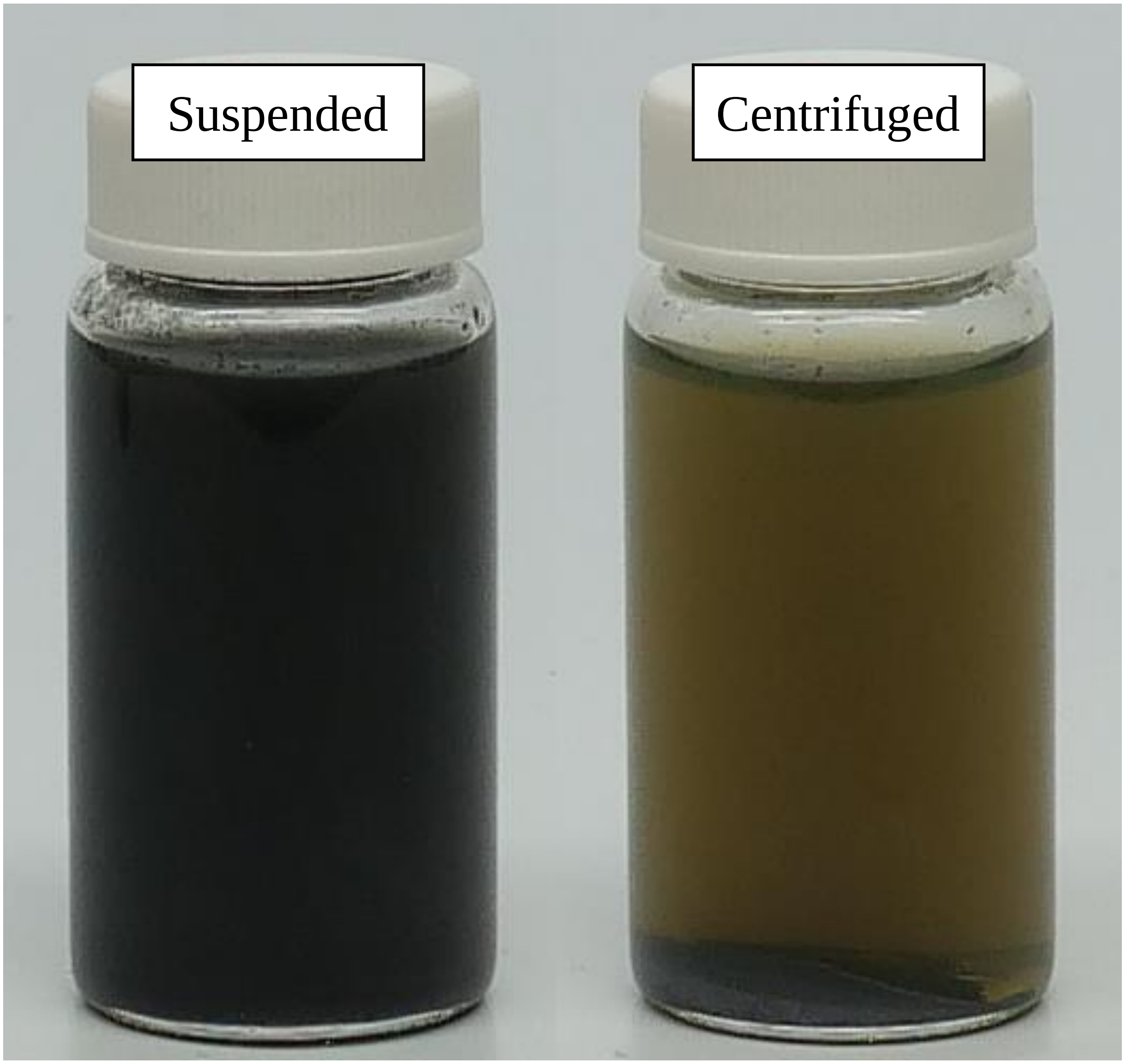

Figure S1. Digital photos of exfoliated MoS_2 suspension in water (left) and layered in water after centrifuging 15 minutes for 4,000 rpm (right).

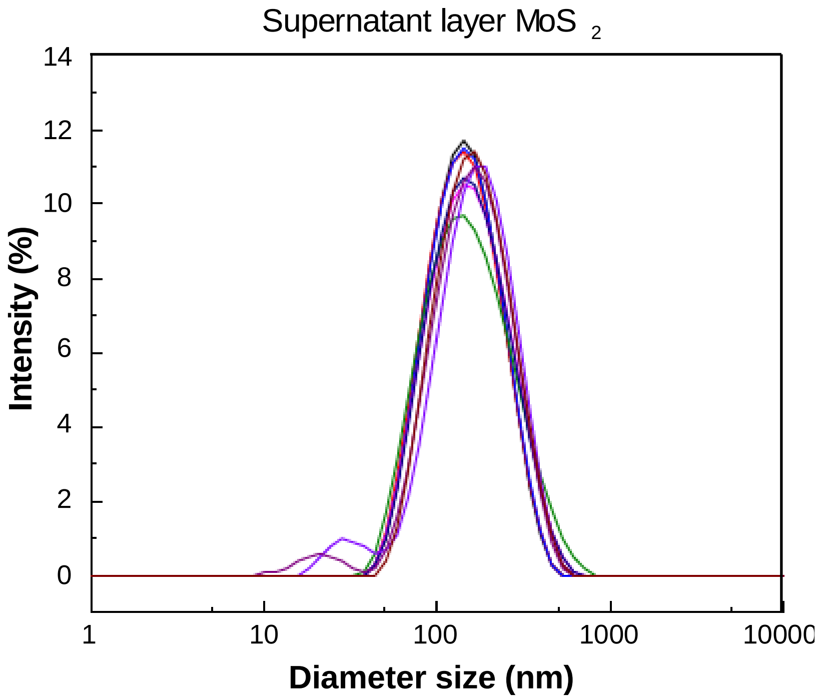

Figure S2. The intensity-averaged particle size (hydrodynamics diameter) distribution of supernatant layer (**Figure S2** right image) from centrifuged exfoliated MoS_2 solution (16.0 mg/mL).

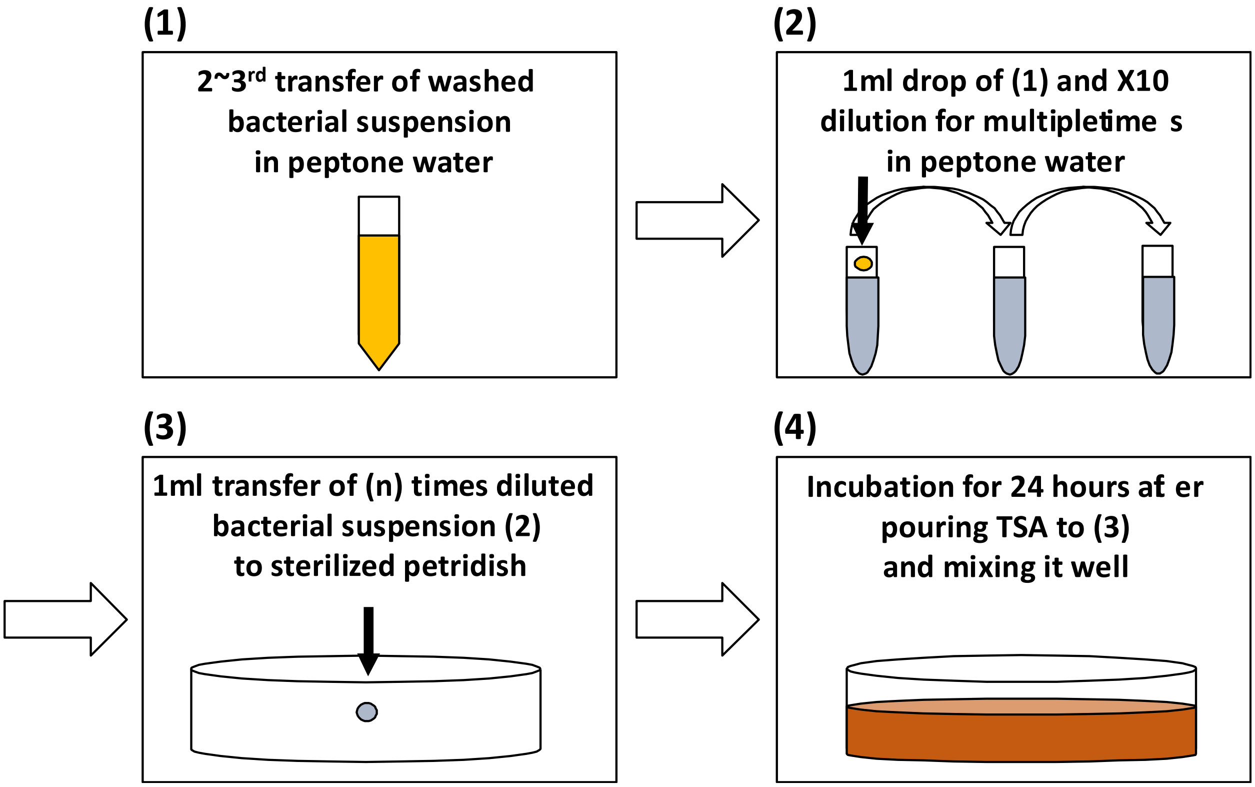

Figure S3. Schematic protocol for preparing the sample for the plate counting methodology. After (4), the number of grown colonies (CFU/mL) are counted within range below 200 and will be multiplied with 10n times.

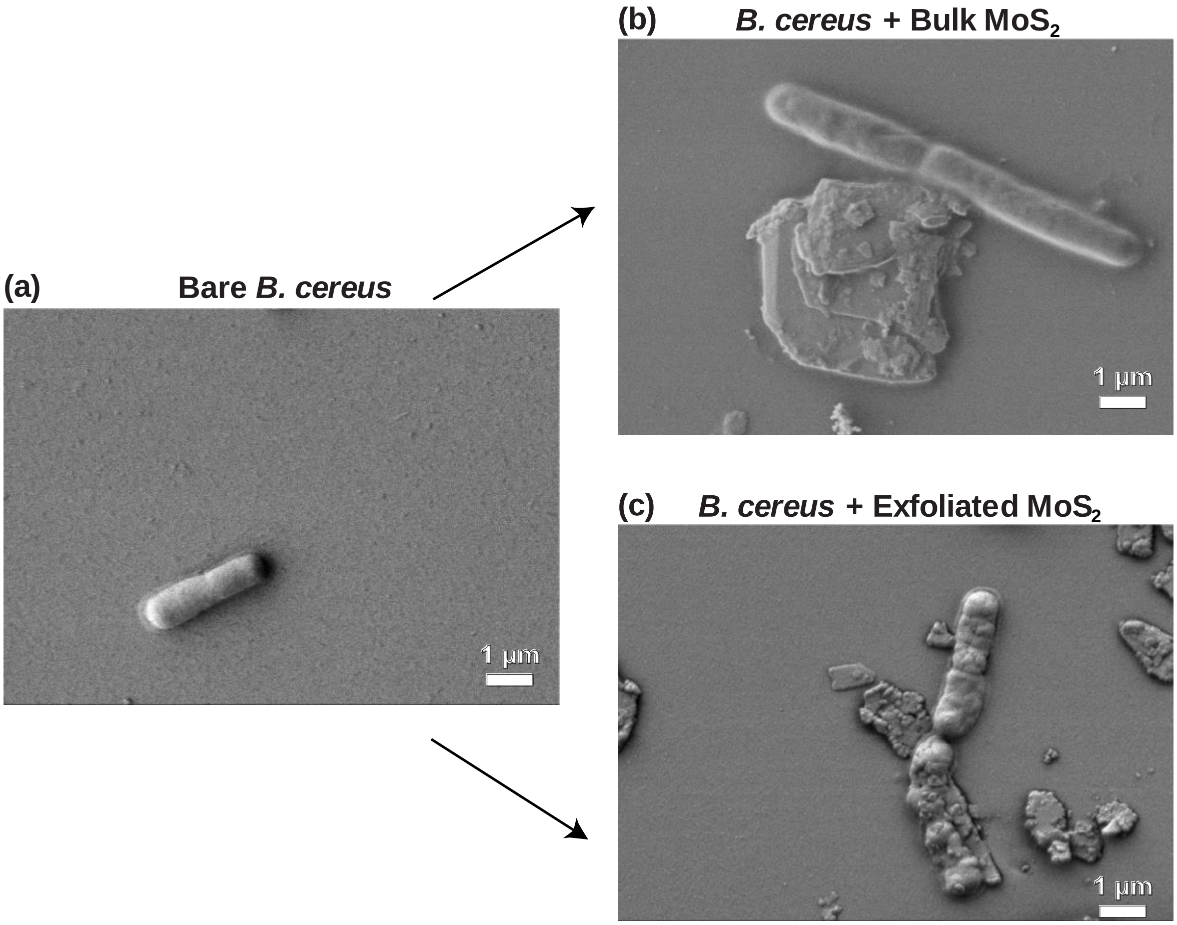

Figure S4. More SEM image showing interactions between soil bacteria and MoS_2 particles (4.0 mg/mL for 12 hours) after they are dried on a silicon wafer surface (a) Bare *Bacillus cereus* (b) *Bacillus cereus* mixed with bulk MoS_2 (c) *Bacillus cereus* mixed with exfoliated MoS_2

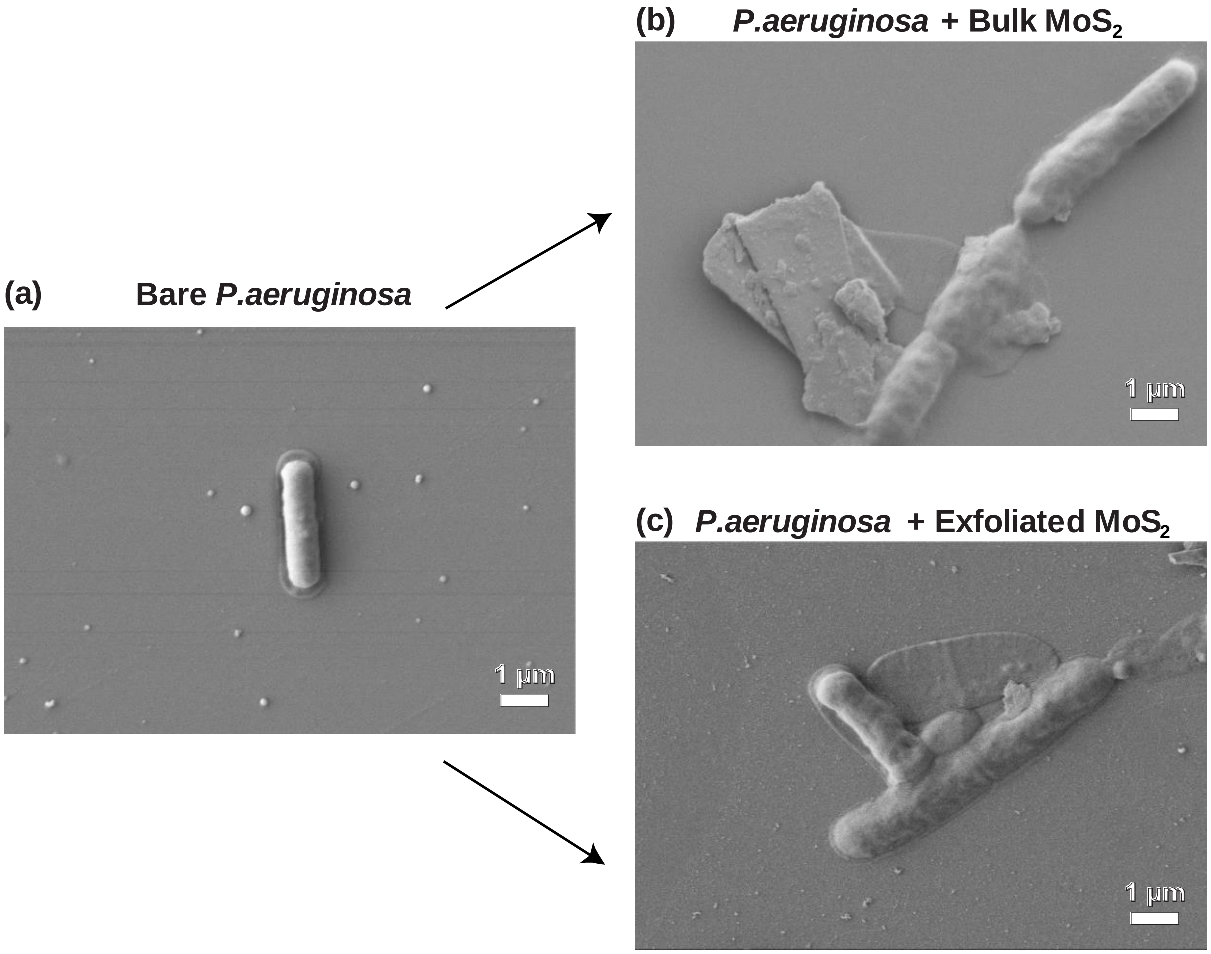

Figure S5. More SEM image showing interactions between soil bacteria and MoS_2 particles (4.0 mg/mL for 12 hours) after they are dried on a silicon wafer surface (a) Bare *Pseudomonas aeruginosa* (b) *Pseudomonas aeruginosa* mixed with bulk MoS_2 (c) *Pseudomonas aeruginosa* mixed with exfoliated MoS_2

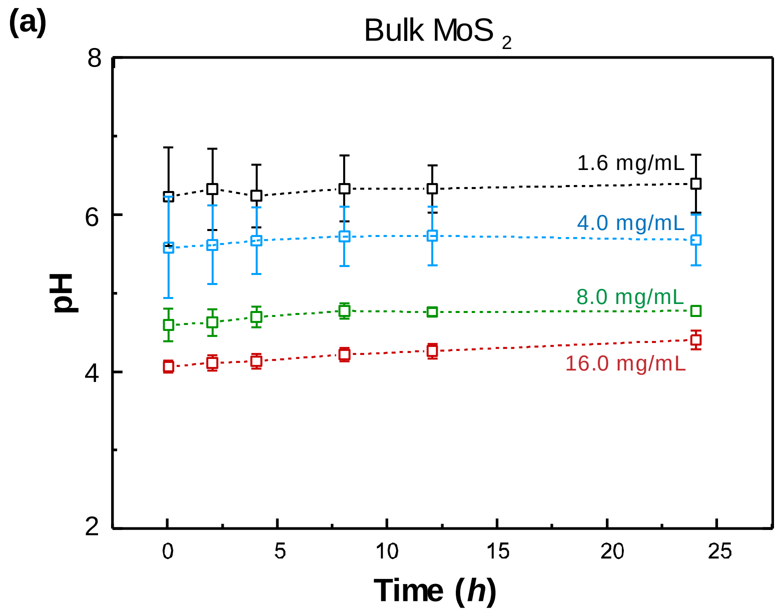

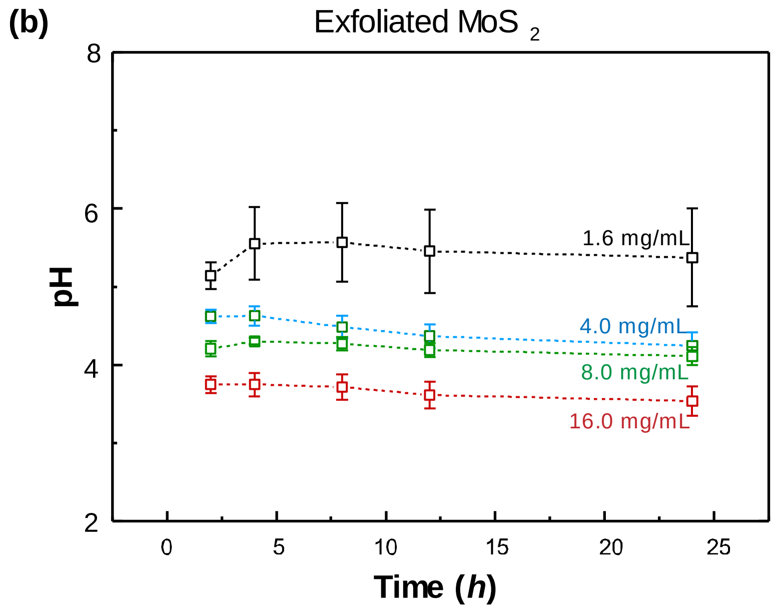

Figure S6. PH measurement for MoS_2 in ultrapurified water stirred with magnetic bar in 24-hour timeline (a) for bulk MoS_2 (b) 1 hour exfoliated MoS_2. The error bars represent standard deviation from the mean.

Table S1. Starting material (MoS_2) information: (**a**) is a product specification offered from the company. (**b**) is bulk MoS_2 EDX result, (**c**) is exfoliated MoS_2 EDX result with freeze dried powder from the sample solution. This X-ray spectroscopy data was measured with Field Emission Scanning Electron Microscope (FE-SEM, JEOL JSM-7500F, Jeol USA, Peabody, MA, USA**).**

(a)

**41827 Molybdenum(IV) sulfide, 99% (metals basis)**	
Product Number	41827
CAS number	1317-33-5
MDL number	MFCD00003470
Molecular formula	MoS_2
Appearance (Color)	Gray to black
Form	Powder
Total Metal Impurities	$\leq$ 1%
Particle Size	-325 mesh

* This information is available at this link (https://alfaaesar.com/en/prodspec/041827)

(b)

**Element**	**Weight%**	**Atomic%**	**Spectrum**
O K	0.00	0.00	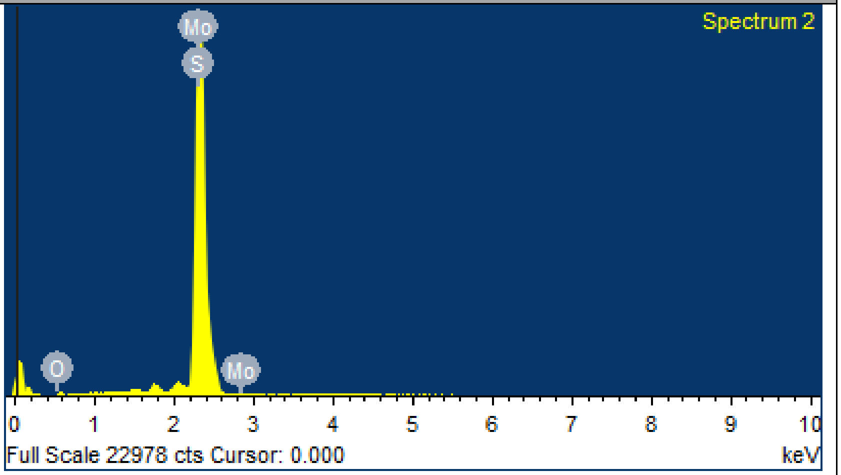
S K	33.51	60.12	
Mo L	66.49	39.88	
Totals	100.00		

(c)

<table>
<tr><th>Element</th><th>Weight%</th><th>Atomic%</th><th>Spectrum</th></tr>
<tr><td>O K</td><td>0.00</td><td>0.00</td><td rowspan="4">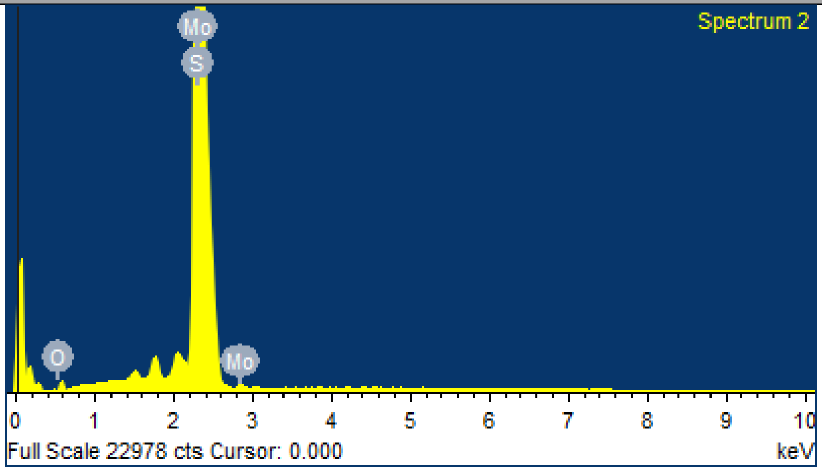
</td></tr>
<tr><td>S K</td><td>31.86</td><td>58.32</td></tr>
<tr><td>Mo L</td><td>68.14</td><td>41.68</td></tr>
<tr><td>Totals</td><td>100.00</td><td></td></tr>
</table>

Table S2. Statistical data derived from Figure 3-4. (**a**) P values for comparing the survival average per different concentrations for each timeline. The higher the p value is, it implies that the data group are more similar. (**b**) Decreased amount of survival average from bulk MoS2 condition to 1 hour exfoliated MoS2 condition

(a)

P value	***B.cereus*** bulk vs 1 hr exfoliated MoS_2	***P.aeruginosa*** bulk vs 1 hr exfoliated MoS_2	**Bulk MoS_2** ***B.cereus*** **vs** ***P.aeruginosa***	**1 hr exfoliated MoS_2** ***B.cereus*** **vs** ***P.aeruginosa***
0hr	Identical; all data start from 1 at 0hr after the normalization			
2hr	0.005449	0.014493	0.006341	0.00989
4hr	0.00048	0.001023	0.000196	0.009816
8hr	0.000625	0.003851	0.003873	0.027004
12hr	0.007226	0.002205	0.017038	0.090325
24hr	0.001784	0.002404	0.039466	0.073406

(b)

Subtraction of normalized survival average at bulk MoS_2 and exfoliated MoS_2	Concentration of the MoS_2	0hr (identical; all data start from 1 at 0hr after the normalization)	2hr	4hr	8hr	12hr	24hr
B.cereus	**1.6 mg/mL**	0.00	0.00	0.44	0.43	0.61	0.31
	**4.0 mg/mL**	0.00	0.05	0.52	0.31	0.32	0.07
	**8.0 mg/mL**	0.00	0.51	0.27	0.05	0.03	0.01
	**16.0 mg/mL**	0.00	0.30	0.23	0.07	0.04	0.00
P.aeruginosa	**1.6 mg/mL**	0.00	0.03	0.07	0.16	0.19	0.22
	**4.0 mg/mL**	0.00	0.39	0.31	0.06	0.00	0.01
	**8.0 mg/mL**	0.00	0.08	0.05	0.06	0.01	0.01
	**16.0 mg/mL**	0.00	0.18	0.08	0.07	0.05	0.01

Table S3. Calculated data table from 8hr dose response curve fitting. The ± values are standard errors calculated by ORIGIN PRO 8.

8hr Dose Response curve fitting data	min	max	$\log_{10}(x_0)$	slope	EC20	EC50	EC80
B.cereus with **bulk** MoS_2	0	100	0.26 ± 0.10	3.58 ± 0.65	1.23 ± 0.36	1.81 ± 0.41	2.66 ± 0.46
B.cereus with **1 hr exfoliated** MoS_2			0.16 ± 0.06	4.24 ± 0.32	1.04 ± 0.16	1.45 ±0.19	2.01 ± 0.23
P.aeruginosa with **bulk** MoS_2			0.00 ± 0.19	2.64 ± 0.84	0.59 ± 0.35	1.00 ± 0.43	1.69 ± 0.45
P.aeruginosa with **1 hr exfoliated** MoS_2			-0.23 ± 0.12	3.36 ± 0.60	0.39 ± 0.13	0.59 ± 0.16	0.89 ± 0.19
